# Functional imaging for regenerative medicine

**DOI:** 10.1186/s13287-016-0315-2

**Published:** 2016-04-19

**Authors:** Martin Leahy, Kerry Thompson, Haroon Zafar, Sergey Alexandrov, Mark Foley, Cathal O’Flatharta, Peter Dockery

**Affiliations:** Tissue Optics & Microcirculation Imaging Group, School of Physics, National University of Ireland (NUI), Galway, Ireland; Centre for Microscopy and Imaging, Anatomy, School of Medicine, National University of Ireland (NUI), Galway, Ireland; Medical Physics Research Cluster, School of Physics, National University of Ireland (NUI), Galway, Ireland; Regenerative Medicine Institute (REMEDI), National University of Ireland (NUI), Galway, Ireland; Chair of Applied Physics, National University of Ireland (NUI), Galway, Ireland

**Keywords:** Microscopy, Imaging, Stem cells, Label-free, Optical coherence tomography, Photoacoustic imaging, Functional

## Abstract

In vivo imaging is a platform technology with the power to put function in its natural structural context. With the drive to translate stem cell therapies into pre-clinical and clinical trials, early selection of the right imaging techniques is paramount to success. There are many instances in regenerative medicine where the biological, biochemical, and biomechanical mechanisms behind the proposed function of stem cell therapies can be elucidated by appropriate imaging. Imaging techniques can be divided according to whether labels are used and as to whether the imaging can be done in vivo*.* In vivo human imaging places additional restrictions on the imaging tools that can be used. Microscopies and nanoscopies, especially those requiring fluorescent markers, have made an extraordinary impact on discovery at the molecular and cellular level, but due to their very limited ability to focus in the scattering tissues encountered for in vivo applications they are largely confined to superficial imaging applications in research laboratories. Nanoscopy, which has tremendous benefits in resolution, is limited to the near-field (e.g. near-field scanning optical microscope (NSNOM)) or to very high light intensity (e.g. stimulated emission depletion (STED)) or to slow stochastic events (photo-activated localization microscopy (PALM) and stochastic optical reconstruction microscopy (STORM)). In all cases, nanoscopy is limited to very superficial applications. Imaging depth may be increased using multiphoton or coherence gating tricks. Scattering dominates the limitation on imaging depth in most tissues and this can be mitigated by the application of optical clearing techniques that can impose mild (e.g. topical application of glycerol) or severe (e.g. CLARITY) changes to the tissue to be imaged. Progression of therapies through to clinical trials requires some thought as to the imaging and sensing modalities that should be used. Smoother progression is facilitated by the use of comparable imaging modalities throughout the discovery and trial phases, giving label-free techniques an advantage wherever they can be used, although this is seldom considered in the early stages. In this paper, we will explore the techniques that have found success in aiding discovery in stem cell therapies and try to predict the likely technologies best suited to translation and future directions.

## Background

A well-chosen imaging technique provides a means to produce high-impact discovery and validation data for the translation of novel regenerative therapies, but choosing the right imaging tool can be tricky and is too often biased by familiarity. Hence we try to provide, in this paper, a means to compare the best known imaging technologies in terms of their capabilities and limitations for stem cell research. Table 1 provides an overview of the optimal stem cell tracking characteristics, the probes used to achieve this, and the appropriate imaging modalities with their advantages and disadvantages. The techniques are discussed in more detail in the following paragraphs.

**Table 1:** An overview of the optimal stem cell tracking characteristics, the probes used to achieve this, and the appropriate imaging modalities with their advantages and disadvantages

Optimal stem cell tracking probe characteristic	Optimal cellular probe	Examples	Probe disadvantages	Imaging modality
Absorbance/emission spectra within “optical window”	Fluorescence	Reporter genes (e.g. iRFP), quantum dots, exogenous probes (e.g. PKH26)	Requires genetic modification and excitation light, high background due to autofluorescence, signal loss with cell division, low depth of imaging, limited spatial resolution	FLI
	Bioluminescence	Reporter genes (e.g. fLuc)	Requires genetic modification and exogenous substrate administration	BLI
	Photoacoustic	Reporter genes (e.g. LacZ, iRFP), endogenous labels (e.g. Hb, melanin)	Requires excitation light and may require genetic modification, expensive equipment	PAI
High signal sensitivity/intensity	Radionuclide	Reporter genes (e.g. hNIS), ^99m^Tc, ^111^In, ^18^F FDG	Ionizing radiation, poor anatomical detail (but can be combined with magnetic resonance or x-ray), radioactive decay limits imaging time, cellular toxicity, may require genetic modification, expensive	SPECT, PET
	Electron density	Gold nanoparticles	Limited spatial/soft tissue resolution, ionizing, not indicative of cell viability, expensive	x-ray, CT
	Fluorescence	As described above	As described above	FLI
	Bioluminescence	As described above	As described above	BLI
	Photoacoustic	As described above	As described above	PAI
High spatial resolution	Magnetic resonance	Iron oxides, microcapsules	Low signal intensity, not indicative of cell viability, expensive	MRI
High temporal resolution/real time tracking	Echography	Microbubbles, perfluorocarbons	Low resolution, acoustic artefacts, subject to user bias	US
	Fluorescence	As described above	As described above	FLI
	Bioluminescence	As described above	As described above	BLI
	Photoacoustic	As described above	As described above	PAI
	Radionuclide	As described above	As described above	SPECT, PET
High imaging depth	Photoacoustic	As described above	As described above	PAI
	Echography	As described above	As described above	US
	Radionuclide	As described above	As described above	SPECT, PET
High cellular retention/signal retention upon cell division	Fluorescence	Reporter genes (e.g. iRFP)	As described above	FLI
	Bioluminescence	As described above	As described above	BLI
	Photoacoustic	As described above	As described above	PAI
High anatomical detail	Magnetic resonance	As described above	As described above	MRI
	Electron density	As described above	As described above	x-ray, CT
	Multimodal systems which include MRI or x-ray			
Low cellular toxicity/non-ionizing	Echography	As described above	As described above	US
	Magnetic resonance	As described above	As described above	MRI
	Fluorescence	As described above	As described above	FLI
	Bioluminescence	As described above	As described above	BLI
Quantifiable signal	Fluorescence	As described above	As described above	FLI
	Bioluminescence	As described above	As described above	BLI
No cellular genetic modification	Echography	As described above	As described above	US
	Radionuclide	^99m^Tc, ^111^In, ^18^F FDG	As described above	SPECT, PET

*BLI* bioluminescence imaging, *CT* computed tomography, *FLI* fluorescence imaging, ^*18*^ *F FDG* fluoro-2-deoxy-d-glucose, *Hb* haemoglobin, ^*111*^ *In* indium, *MRI* magnetic resonance imaging, *PAI* photoacoustic imaging, *PET* positron emission tomography, *SPECT* single photon emission computed tomography, ^*99m*^ *Tc* technetium, *US* ultrasound

## Main text

### Overview of functional imaging for regenerative medicine

Functional imaging, especially when provided in its structural context, provides a platform for all branches of regenerative medicine research. The technology is constantly being advanced to image faster, deeper, less invasively, and more quantitatively, driving discovery of both biological and clinical mechanisms. This article will review some of the plethora of advances that have been made in recent years in technologies that have enabled discovery in the field of stem cell research. Topics such as in vivo fluorescence imaging and the benefits of label-free techniques such as optical coherence tomography (OCT) and photoacoustic imaging (PAI) will be discussed, along with super resolution microscopy and radionuclide imaging.

### Stem cell imaging in regenerative medicine

Stem cells have the ability to undergo clonal expansion and to differentiate into multiple cell types; adult stem cells offer advantages over embryonic stem cells due to their ease of isolation and lack of ethical issues [1]. Regenerative medicine, or the use of stem cells as therapies, consists of multi-disciplinary approaches with the aim of restoring function to diseased tissues and organs. Such cell-based therapies have been extensively investigated as promising avenues of treatment for a host of disease types, including, but not limited to, cardiac disease, diabetes and orthopaedics. For the current rate of progress to be maintained, non-invasive and reproducible methods to monitor and assess stem cell integration and survival in disease models are of paramount importance. Imaging techniques with high spatial and temporal resolution will enable accurate tracking of transplanted stem cells to disease loci in vivo over a long period of time in pre-clinical (animal) models and, ultimately, in clinical trials. Information obtained from such studies will also allow scientists and clinicians to optimise stem cell administration regimens (e.g. dose, route of administration, timing) and to assess the efficacy of a cell-based treatment.

Currently, tracking stem cell migration and engraftment is achieved using appropriate imaging systems in parallel with endogenous and exogenous cell-labelling methods. An ideal cellular label should:

- be biocompatible and non-toxic to cells;
- be quantifiable;
- be inexpensive;
- remain undiluted following cell division;
- not leak into adjacent non-transplanted cells;
- remain stable over long periods of time in vivo;
- not interfere with normal cell function;
- not require genetic modification or the injection of a contrast agent.

Stem cells can be genetically modified to express reporter genes or proteins that can emit fluorescence/bioluminescence (or other useful proteins such as lacZ or NIS) or be treated to uptake exogenous contrast agents, such as organic dyes, nanoparticles, radionuclides, or magnetic compounds [2].

### In vivo fluorescence imaging

The collection of data from an innate biological site is one of the largest advantages of in vivo imaging of any form. Macroscopic imaging of either animal or human sources, as opposed to the imaging of tissue explants or cells from culture, encounters an array of complications. In vivo fluorescence imaging is similar to conventional fluorescence microscopy in that high-end low-light cameras are used to detect an emission signal generated from a fluorophore or probe [3, 4]. In recent years, the development of stem cell therapies for treatment of a vast array of diseases has progressed rapidly [5]. Molecular tagging and the addition of probes to monitor, track, and assess the administered cells in a non-invasive manner in vivo, in both animal and human clinical studies, will be discussed in this section. Further to this, the use of multimodal approaches (fluorescence in conjunction with bioluminescence and high-resolution imaging techniques) will be briefly highlighted.

Ex vivo histopathological analysis of modified stem cell behaviour was traditionally carried out, using fluorescent probes, on excised biopsies from animal model studies. These examinations were incapable of providing real-time information about alterations to the tissues under study. Despite this limitation, these probes provided the framework for many of the newer generations of markers currently in use today to be developed and refined. The incorporation of reporter genes into cellular machinery has provided scientists with a method to visualise cells, via fluorescent modifications, to a depth of about 2 mm into the tissue. The incorporation of these genes into a cell is referred to as indirect labelling. Reporter genes allow the monitoring of physiologically relevant biological processes as they occur in situ. Traditionally, green fluorescent protein (GFP) tags were used in fluorescence imaging to identify cells [6]. The main advantage of this form of labelling is that expression of the functional reporter probe only occurs after the cell has transcribed the gene of interest and the mRNA is translated into the modified version of the protein and a biosensor is created. This allows direct correlations to be drawn between the levels of expression of the probe and cell viability. The expression of the modified gene is propagated to future generations of cells and, in this way, the longevity of this method is preferable in an in vivo scenario as it would potentially create a long-term reporter of cell stem functionality and enable tracking/tracing over a lengthier period of time. Genetic modification of cells, via transfection (non-viral vectors) or transduction (viral vectors), that are employed in order to allow the incorporation of these reporter genes is, at present, the major limiting factor of this technique [7]. The long-term safety of incorporating transformed genetic material and the potential for immune responses or tumour development in recipients of these therapies requires further investigation and regulation at a clinical trial level. With a strong focus on safety and therapeutic efficacy for stem cell delivery, many laboratories are developing alternative methods to allow the integration of reporters into the cellular genome [8]. Recent work has focused on the development of fluorescent probes for incorporation in reporter genes amongst other uses. Fluorescent probes whose spectra are in the far red, towards the near infrared (NIR) portions of the spectrum of light (650–900 nm), are experimentally the most desirable for scientists wishing to carry out in vivo imaging. The potential for alterations to the physiological state of the cell under study must be monitored when utilising any type of fluorescence imaging technique. The benefits of imaging in this portion of the spectrum will be discussed in later sections. Earlier probe variants including mKate, with excitation and emission at 588 and 635 nm and synthesised from the sea anemone *Entacmaea quadricolor*, were developed for whole body imaging, and more recently phytochrome (photosensor) from the bacteria *Deinococcus radiodurans* has allowed production of the IFP 1.4 marker [9, 10]. Despite these advances, quantum yield for these probes remained poor. Newer probes including iRFP (near-infrared fluorescent protein) are aimed at increasing the fluorescence output and signal intensity through modifications of these phytochromes, and display improved pH and photo-stability in vivo [11]. The use of optogenetics, or the control of biological processes in mammals (both cells and tissues) by light, is emerging as a very powerful manipulation technique. This method combines the genetic modifications discussed above, with the possible inclusion of NIR probes, and the potential to act as a therapy mediator for stem cell treatments [12, 13]. Work to date has concentrated on mainly neural stem cells in animal models [14, 15].

The combination of fluorescence, bioluminescence, and high-resolution probes are referred to as multimodal reporter probes. The combination of the best aspects of all probes and techniques allows a much great amount of data to be collected from one source. Recent work from Roger Tsien’s group has shown that one of these triple modality reporters has been implemented in an in vivo animal study for qualitative tumour therapy and efficacy of drug delivery [16]. The development and advancement in the engineering and construction of these fluorescent and multimodal probes holds most hope for successful deep tissue in vivo fluorescence imaging.

In summary, fluorescent imaging modalities are simpler, cheaper, more user friendly, and convenient to carry out than their higher resolution counterparts. The development of high-sensitivity cameras, which are capable of detecting very low levels of gene expression, and the quantitatively close relationship between cell number and fluorescence detection signals are all major benefits of these techniques.

### The advantages of label-free optical imaging techniques

Appropriate imaging modalities are needed for the tracking of stem cells to investigate various biological processes such as cell migration, engraftment, homing, differentiation, and functions. The ideal modality for tracking stem cells requires high sensitivity and high spatial resolution, non-toxic imaging. Contrast agents should be biocompatible and highly specific to reduce perturbation of the target cells. The ideal modality should provide non-invasive, depth-resolved imaging in situ and be able to detect single cells, and should show a difference between cell loss and cell proliferation. Currently none of the known imaging modalities has all of these characteristics [17, 18].

In contrast to the above-mentioned modalities, this section will focus on those techniques which do not employ the use of an endogenous/exogenous contrasting agent. Label-free imaging techniques provide the unique possibility to image and study cells in their natural environment.

For example, such techniques can be used for the isolation of human pluripotent stem cells (hPSCs), enriched to 95–99 % purity with >80 % survival, and to keep normal transcriptional profiles, differentiation potential, and karyotypes [19]. Well-known label-free imaging modalities, such as quantitative phase microscopy (QPM), are used to reconstruct nanoscale phase information within cells, including living cells [20]. Interference reflection microscopy (IRM), also sometimes referred to as Interference Reflection Contrast, or Surface Contrast Microscopy, is often used in conjunction with QPM [21]. This non-invasive label-free technique is employed in the study of cellular adhesions, migration, cell mitosis, and cytotoxicity amongst other parameters in stem cell cultures such as human induced pluripotent stem cells (hIPSCs). Greyscale images are created from the slight variations generated in optical path differences where reflected light is used to visualise structures which are at, or near, a glass coverslip surface [22]. This technique can provide quantitative information on the intracellular cytoplasmic and nuclear alterations often required by scientists whilst assessing stem cells and their differentiation state in culture, and therefore assist in the screening selection of hIPSC colonies [21]. Optical diffraction tomography permits three-dimensional (3D) image reconstruction of a single cell [23–25]. The oblique-incidence reflectivity difference (OI-RD) microscope was proposed for label-free, real-time detection of cell surface markers and applied to analyse stage-specific embryonic antigen 1 (SSEA1) on stem cells in the native state [26]. Another imaging modality, digital holographic microscopy (DHM), provides the possibility for imaging of a 3D volume with a single exposure which is very useful for imaging of living cells. DHM was combined with light scattering angular spectroscopy to provide spatially resolved quantitative morphological information [27–29], improved resolution via a synthetic aperture approach [30–32], and used for 3D tomographic imaging [33]. The disadvantages of these techniques are that they are not depth-resolved and cannot be applied to highly scattered media like tissue, or they are too slow and not suitable for in vivo applications.

The recently developed spectral encoding of the spatial frequency (SESF) approach provides the means for label-free visualization of the internal submicron structure in real time with nanoscale sensitivity [34, 35], which could be a good alternative for in vivo stem cell investigation. Precise characterisation of the internal structure with nanoscale accuracy and sensitivity can be performed using the spectral distribution of scattered light to reconstruct the nanoscale structural characteristics for each pixel [36]. The theoretical basis for tomographic imaging with increased spatial resolution and depth-resolved characterization of the 3D structure has been established [37]. Label-free, depth-resolved structural characterization of highly scattering media (tissue, skin) with nanoscale sensitivity, based on the SESF approach, has been proposed [38, 39]. Label-free, super-resolution imaging using the SESF approach has been demonstrated recently [40]. The parallel development of label-free imaging techniques and the use of new non-toxic contrast agents are very encouraging.

### Optical coherence tomography for study of the stem cells

OCT is one of the promising techniques for depth-resolved imaging of biomedical objects. OCT, developed in 1991 by Fujimoto and co-workers at Massachusetts Institute of Technology [41], can be considered as an optical analogue of the ultrasound technique. In comparison with ultrasound, OCT provides improved resolution of depth-resolved images to microscale, but the penetration depth is limited. OCT can provide unique depth-resolved morphologic and functional information. For example, OCT facilitates cellular level structural and functional imaging of living animals and human tissues [42–44], performs vibration measurements in the retina and ear at the nanoscale [45, 46], and depth-resolved imaging of the cornea and mapping of vasculature networks within human skin [47–51]. OCT has also received much attention in the field of tissue engineering [52–54]. In contrast to confocal microscopy, two-photon microscopy, and other optical depth-resolved imaging techniques, OCT provides a much better penetration depth: about 2 mm in tissue instead of 100–500 microns. Recently, OCT (the standard spectral radar-OCT (SR-OCT) system (Model OCP930SR; Thorlabs Inc., Newton, NJ, USA)) has been applied as a new imaging strategy to investigate planarian regeneration in vivo in real time [55]. The signal attenuation rates, intensity ratios, and image texture features of the OCT images were analysed to compare the primitive and regenerated tissues, showing that they might provide useful biological information regarding cell apoptosis and the formation of a mass of new cells during planarian regeneration.

**Fig. 1:**
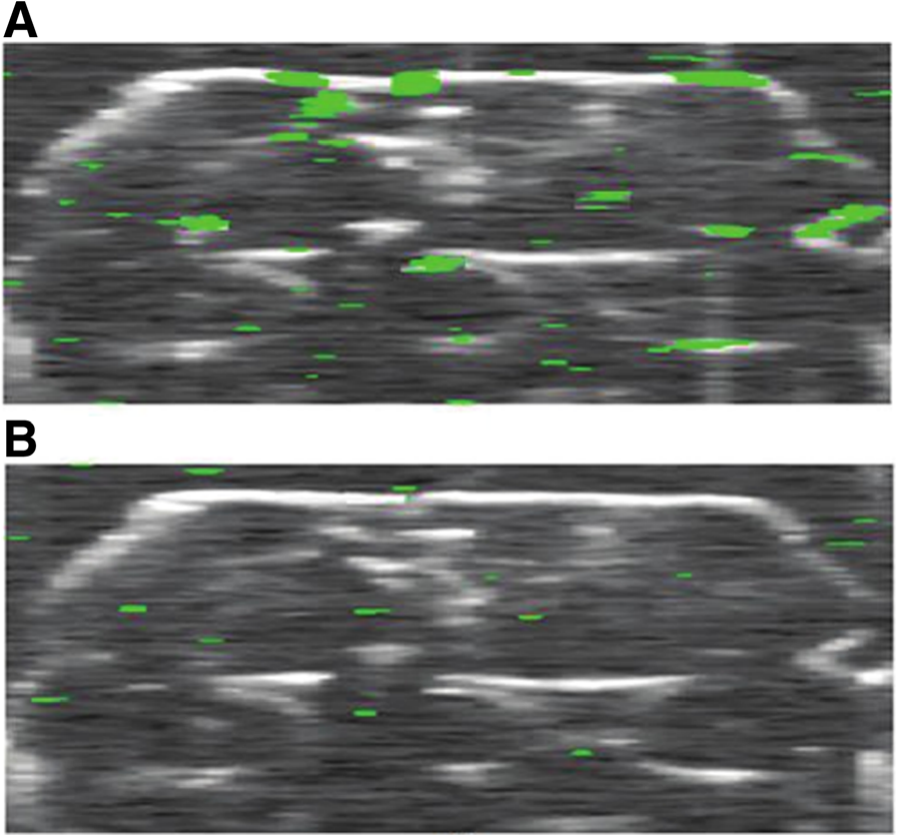
**a** Combined structural and photothermal image of the scaffold seeded with SWNT-loaded MSCs with the laser turned on. **b** Combined structural and photothermal image of the scaffold seeded with SWNT-loaded MSCs with the laser turned off

The spatial resolution of conventional OCT systems is limited to about 10 microns and is insufficient for cell imaging. Only some specific complicated systems—optical coherence microscopes (OCMs; http://www.rle.mit.edu/boib/research/optical-coherence-microscopy), such as high-definition OCT (HD-OCT) and micro-OCT—provide micrometre resolution in both transverse and axial directions in order to visualise individual cells (Skintell; Agfa Healthcare, Mortsel, Belgium) [56]. This system uses a two-dimensional, infrared-sensitive (1000–1700 nm) imaging array for light detection and enables focus tracking along the depth of the sample. The movements of the focal plane and the reference mirror are synchronised. As a result, the lateral resolution is 3 μm at all depths of the sample. Together with limited resolution, OCT provides only limited molecular sensitivity. To solve the problem, application of OCT for stem cell research is based on using extrinsic contrast agents such as magnetic and iron oxide particles, proteins, dyes, various types of gold nanoparticles, carbon nanotubes, and so forth. For example, the first report to demonstrate the feasibility of photothermal optical coherence tomography (PT-OCT) to image human mesenchymal stem cells (hMSCs) labelled with single-walled carbon nanotubes (SWNTs) for in vitro cell tracking in 3D scaffolds has been presented recently [57]. A photothermal BMmode scan was performed with excitation laser driving with a frequency of 800 Hz. Figure 1a shows the cross-sectional image of the combined structural and photothermal signal of the scaffold seeded with SWNT-loaded MSCs with the photothermal excitation laser turned on. Figure 1b shows the corresponding image with the excitation laser turned off. It was shown that PT-OCT imaging together with the SWNT nanoprobes looks promising for visualising and tracking of MSCs in vitro and in vivo.

Another possibility is multimodal imaging, which may minimise the potential drawbacks of using each imaging modality alone [17], such as the combination of OCT and other imaging techniques (confocal microscopy, dielectric spectroscopy (DS), fluorescence microscopy, and so forth) [56–60]. Bagnaninchi [58] used a spectral domain optical coherence tomography (SDOCT) combined with DS to qualitatively assess adipose-derived stem cells loaded in 3D carriers. The broadband (from 20 MHz to 1 GHz) DS spectra were acquired at high cell concentration simultaneously with 3D OCT imaging. Chen et al. [59] used high-resolution OCT to visualise the microstructures of the engineered tissue scaffolds in 3D and to investigate the key morphological parameters for macroporous scaffolds, while fluorescence imaging was conducted to monitor the population of labelled hMSCs loaded on to the surface of the scaffolds. Ksander et al. [60] used confocal microscopy, multiphoton microscopy and OCT to study the conditions for limbal stem cell maintenance, and corneal development and repair. Lathrop et al. [61] showed, using a combination of OCT and confocal microscopy, that OCT successfully identified the limbal palisades of Vogt that constitute the corneal epithelial stem cell niche, and offered the potential to assess and intervene in the progression of stem cell depletion by monitoring changes in the structure of the palisades. Schwartz et al. [62] used SDOCT together with visual field testing, slit-lamp biomicroscopy, ophthalmoscopy, fluorescein angiography, autofluorescence imaging, fundus photography, and electroretinography to study human embryonic stem cell-derived retinal pigment epithelium in patients with age-related macular degeneration and Stargardt’s macular dystrophy. The results provide evidence of the medium- to long-term safety, graft survival, and possible biological activity of pluripotent stem cell progeny in individuals with any disease, and suggest that human embryonic stem-derived cells could provide a potentially safe new source of cells for the treatment of various unmet medical disorders requiring tissue repair or replacement.

A potential alternative to using contrast agents is the recently developed nano-sensitive OCT which increases sensitivity to structural alterations in space and in time by more than 100 times [38, 39].

### Optical coherence phase microscope

In 2011, Bagnaninchi’s group demonstrated that live stem cells could be differentiated from their surrounding environment by mapping the optical phase fluctuations resulting from cellular viability and associated cellular and intracellular motility with an optical coherence phase microscope (OCPM) [63], an OCT modality that has been shown to be sensitive to nanometer-level fluctuations. In subsequent studies [64, 65], they examined murine pre-osteoblasts and human adipose-derived stem cells growing within two distinct polymeric constructs: 1) a 3D printed poly(d,l-lactic-co-glycolic acid) fibrous scaffold; and 2) hydrogel sponges (alginate). In addition to providing cell viability information, the endogenous contrast between cells and scaffolds generated by cellular motility enabled real-time, label-free monitoring of 3D engineered tissue development [65].

### Photoacoustic imaging

PAI (less often called optoacoustic imaging) is an emerging biomedical imaging technique that exploits laser generated ultrasound (US) waves to generate 3D images of soft tissues. Tissue is exposed to pulsed nanosecond laser light, resulting in localised heating of the tissue. The increase in temperature of few degrees milliKelvin causes transient thermoelastic tissue expansion which generates broadband (MHz) pressure waves. The ultrasonic waves created are then detected using wideband transducers and further converted into images. PAI is a hybrid imaging modality that combines the high contrast and spectroscopic-based specificity of optical imaging with the high spatial resolution of US imaging [66]. It provides an integrated platform for functional and structural imaging, which is suitable for clinical translation.

PAI breaks through the optical diffusion limit [67] and provides real-time images with relatively high spatial resolution, without ionizing radiation being involved. The key advantages of the PAI technique over other imaging modalities include:

- the detection of haemoglobin, lipids, water, and other light absorbing molecules with higher penetration depth than pure optical imaging techniques;
- the ability to provide tissue information using an endogenous contrast alone [68];
- the imaging of optical absorption with 100 % sensitivity, which is two times greater than those of OCT and confocal microscopy;
- unlike ultrasonography and OCT, it is speckle-free [69] and provides inherently background-free detection.

**Fig. 2:**
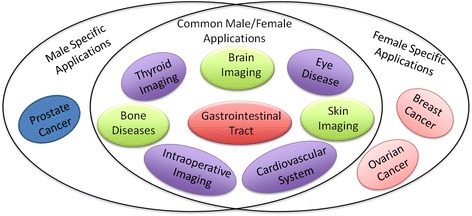
An overview of potential clinical applications of PAI

The development of PAI techniques continues to be of substantial interest for clinical imaging applications in oncology, including screening, diagnosis, treatment planning, and therapy monitoring [70, 71]. PAI-based routines have also been extensively used in accurate determination of metabolic rate during early diagnosis and treatment of various skin and subcutaneous tissue disorders. The other potential implications of PAI encompass the domains of dermatology [72, 73], cardiology [74, 75], vascular biology [76, 77], gastroenterology [78, 79], neurology [80–82], and ophthalmology [83, 84]. Figure 2 summarises the potential clinical applications of PAI.

In PAI, stem cells are typically labelled using biocompatible materials with optical properties such as gold (Au) nanoparticles (NPs) or Au nanorods (NRs). In a recent study, hMSCs were labelled with 20-nm Au NPs before their incorporation into PEGylated fibrin gel [85]. After injecting the fibrin gel intramuscularly into the lateral gastrocnemius (lower limb) of an anaesthetised Lewis rat, PAI was performed to visualise the in vivo neovascularisation and differentiation of hMSCs.

Au NRs have plasmon resonance absorption and scattering in the NIR region, which makes them attractive probes for PAI [86]. In another study, hMSCs were labelled and imaged by silica-coated Au NRs (SiGNRs) [87]. The researchers found that the cellular uptake of SiGNRs can be dramatically increased (fivefold) by silica coating without changing function and viability of hMSCs.

### Microcirculation imaging

Several techniques, including OCT and PAI, can be used to image microcirculatory function. The microcirculation is the usual route for delivery of stem cells by systemic or local intravascular injection. It is also affected by the stem cell therapies which may stimulate or suppress angiogenesis and will often have a major role in regeneration. In addition to the 3D techniques discussed in detail here, several other techniques are available to investigate the microcirculatory response to stem cell therapy, e.g. laser doppler, laser speckle, tissue viability imaging (TiVi), and side stream dark field microscopy [88].

### Confocal reflectance microscopy

Confocal reflectance microscopy employs innate alterations in the refractive index of biological samples to create contrast within an image. Intracellular organelles and protein-protein interactions between these components, or even the interface between two different cell types as would be evident in an epithelial stromal interface, would contribute to contrast variation [89]. In recent years this technique has been used to non-invasively study skin biopsies, myelinated axons, and gather information from the excised bone marrow stem cell niche [90–92]. A combination of both fluorescent and reflectance images can be captured through the installation of a beam splitter into the light path, which allows reflected light from the sample to pass into the detection unit. In highly scattering tissues, like skin, the advantages of confocal microscopy can be combined with OCT techniques to produce the optical coherence microscope (OCM). In this way, higher numerical aperture lenses and coherence gating allows the collection of clearer images through a greater depth in tissues, when compared to either OCT or reflectance confocal modalities alone [93].

### Super-resolution microscopy (nanoscopy)

Sub-cellular imaging, for example of organelles, requires diffraction-unlimited ‘super-resolution’ techniques. True super-resolution is only achievable with near-field optical techniques such as near-field scanning optical microscopy and 4π microscopy. However, mainstream functional super-resolution microscopy or nanoscopy uses the ability to switch fluorescent molecules on and off in a spot size smaller than the Abbé limit to overcome the diffraction limit for image resolution. Fluorescent molecules become “bleached” for some period of time once they have emitted a fluorescent photon. In stimulated emission depletion (STED), the illumination (excitation) spot remains diffraction-limited, but a concentric de-excitation doughnut-shaped beam turns off fluorescence in most of that spot [94]. Since the illumination wavelength is filtered out, only the longer fluorescent wavelength is detected or visible in the microscope. Hence, the smaller the spot at the centre of the doughnut which is allowed to fluoresce, the smaller the spot which can be imaged. Thus, the technique gets around the Abbé limit rather than breaks it. The size of the spot which can be imaged is only limited by the intensity of the doughnut-shaped beam. As this intensity gets larger (GW/cm^2^ have been used), the size of the spot from whence fluorescence can be emitted gets smaller. STED and reversible saturable optical linear fluorescence transitions (RESOLFT) nanoscopy has been found especially useful for neurons or fixed cells and can be used in fast processes [95].

Some other techniques like photo-activated localization microscopy (PALM) and stochastic optical reconstruction microscopy (STORM) tackle this problem statistically [95]. These techniques find the locus of a molecule by fitting a Gaussian profile to the emission. If enough photons are collected, the locus can be identified with an uncertainty less than the diffraction limit. Conversely, two molecules within the lateral optical resolution can only be localised if the emitted photons occur at different times. Thus, these techniques are more suited to slower processes.

PALM, STORM, and STED share the need to switch off molecules and are essentially limited to imaging fluorophores or objects which are labelled with fluorophores which are generally toxic. Nonetheless, there are now well-established methods for labelling almost anything (typically cells or cell components) with fluorescent molecules. They also share the further steps of identification and localization [96]. Ultimately, of course, they are limited by the size of the fluorescent molecule and practical considerations such as the integrity, viability, and drift of the sample. With samples bigger than an individual cell, refractive index variations will cause distortions which are significant on the nanoscale.

### Microcomputed tomography

We are all familiar with the extraordinary imaging capabilities of x-ray computed tomography (CT) in the hospital. However, the resolution is limited to approximately 1 mm in favour of penetration depth of tens of centimetres. With higher x-ray dose per voxel, the signal to noise ratio can be sufficient to achieve sub-micron resolution in engineering materials after several hours, although this dose would be too great for living cells and tissues. In vivo microCT uses a small sample bore typically sufficient for a mouse and can generate exquisite structural images with approximately 100-μm resolution in all directions. MicroCT application to stem cell research has already been reviewed by Boerckel et al. in this series [97].

### Radionuclide imaging

Adding the functional capabilities provided by positron emission tomography (PET), PET-CT, and single-photon emission computed tomography (SPECT) imaging allows the stem cell functions to be put in their proper structural context. The earliest studies utilising the tracer principle [98], the use of small amounts of radionuclides in subjects, can be traced back to the 1920s [99]. However, it was development of the sodium iodide (NaI(Tl)) scintillation camera in the 1950s by Hal Anger [100] which was the bedrock of clinical nuclear medicine imaging systems for many decades. In the last decade there has been significant progress made in the development of various pre-clinical imaging systems across many modalities, and SPECT has become one of the principle tools [101, 102]. Several groups, including our own, have been demonstrating the capabilities of new SPECT system configurations [103–107]. Research innovation in this field has been significant with developments in aspects such as image reconstruction, collimation, detection, dual isotope imaging, and multimodality systems. Small animal SPECT (and PET) systems are exquisitely sensitive, capable of measuring picomolar concentrations of radiolabelled biomolecules in vivo with sub-millimetre resolution.

In terms of applications, there is considerable interest in methods where the radiation source is inside the subject and therapeutic applications are mediated by the human sodium iodide symporter (NIS). Several groups have evaluated the potential for the introduction of NIS expression to support imaging and treatment for various cancer types. For example, MSCs can be engineered to express NIS and then home to the tumour site for delivery of therapy [108]. SPECT imaging using ^123^I or ^99m^Tc can be used to confirm the migration of the MSCs to the tumour site, and then ^131^I can be used for therapy.

During the last 10–15 years, small animal radionuclide imaging has undergone rapid technological development and improvement in image performance metrics. Innovations in several areas currently under investigation by several groups will lead to further improvements in the future, and radionuclide imaging will continue to play a vital role in future molecular imaging applications. The development of hybrid imaging with modalities such as PET/CT, PET/MR, SPECT/CT, and, possibly in the near future, SPECT/MR will enable biologists to observe processes in varying time windows from minutes to weeks.

Stem cell tracking requires high spatial resolution and sensitivity. Given that each imaging technique presents its unique set of advantages and disadvantages, the selection of an appropriate imaging modality depends on the application, the goal of the experiment, the subject under study, and so forth. No imaging technique is perfect in all aspects. Optical imaging techniques offer many distinctive advantages such as non-invasiveness, resolution, high spatial and temporal sensitivity, and adaptability, but these techniques are limited by relatively poor tissue depth. Radionuclide imaging has a fair sensitivity (10^–8^ to 10^–9^ μm/L), but it is not suitable for long-term cell tracking due to radioisotope decay. Fluorescence imaging has very high sensitivity (10^–12^ to 10^–15^ μm/L), but this technique is constrained by relatively shallow tissue depth [17]. An overview of the advantages and disadvantages of each technique is presented in Table 2.

**Table 2:** Advantages and disadvantages of techniques listed in the manuscript

Technique	Advantages	Disadvantages
In vivo fluorescence imaging	• Simple, cheap, user friendly techniques • High spatial resolution (~200 nm in x,y,) with high sensitivity cameras • Development of FarRed and NIR probes allow greater tissue visualization with much less damage whilst imaging • High sensitivity (10^–12^ to 10^–15^μm/L)	• Use of a probe generally required which may have repercussions on stem cell physiology • Photo-toxicity to tissue and depth resolved imaging still an issue • Vectors employed to introduce reporter genes are still under scrutiny for safety and efficacy of use in clinical trials
QPM	• Accurate quantitative visualisation of phase changes within cells	• No depth-resolving capabilities
ODT	• Depth-resolving capabilities, resolution of up to 1 μm	• Low penetration depth (a few hundred microns), not suitable for real-time imaging (slow techniques)
DHM	• Imaging of a 3D volume with a single exposure, structural and phase imaging, and also flexibility for image processing. Resolution almost as in conventional microscopy	• Relative complexity (more complicated optical set up), limitation on coherent properties of the light source, on environmental conditions (vibrations, etc.)
SESF and srSESF	• High (nano-scale, ~10 nm demonstrated) sensitivity to structural alterations within object and super- resolution imaging	• More complicated optical set up, for example for detailed quantitative analysis of the structure an imaging spectrometer or swept light source is needed
OCT	• Improved image resolution (morphological and functional information) of depth-resolved images • Can be combined with other imaging techniques for multimodal imaging • Suitable for clinical translation	• Penetration depth is limited ~2 mm into tissue • Spatial resolution is typically limited to ~10 μm, making this technique unsuitable for cell imaging • Limited molecular sensitivity of tissue
OCM	• Enhanced penetration depth compared to standard confocal microscopy; dramatically improved resolution over OCT imaging (up to 1 micron)	• Small penetration depth (compared with OCT)
nsOCT	• Depth-resolved images with high sensitivity (~30 nm demonstrated experimentally)	• Resolution and penetration depth are approximately the same as conventional OCT
OCPM	• Quantitative phase information with high sensitivity, useful for 3D intracellular imaging	• Small depth of field
PAI	• Capable of collecting molecular and spatial information from the tissue using endogenous contrast alone • Greater sensitivity than OCT and confocal imaging • Suitable for clinical translation • The ratio of the imaging depth to the best spatial resolution is roughly a constant of 200	• Sometimes requires the use of biocompatible labelling materials such as gold or silver nanoparticles
Confocal reflectance microscopy	• High spatial resolution images achievable (diffraction limited ~200 nm) • Can work in combination with other modes of microscopy including fluorescence and OCT	• Lack of specific light reflecting probes for confocal microscopy when used in reflectance mode
Super-resolution microscopy (nanoscopy)	• Images created have a higher spatial resolution that normal diffraction limited techniques. (STED x.y resolution ~20–100 nm, PALM and STORM x.y ~20–50 nm) • Increased localization and clarity of intracellular structures due to increased resolution	• Fluorophores or fluorescent markers must be used. Potential for photo bleaching of the sample under study • Expensive equipment • Currently most super resolution techniques are not suitable for live cell imaging • Refractive index variations in the substrate can cause distortions which when translated to the nanoscale can be significant
Microcomputed tomography	• Can generate defined structural images with increased all round resolution (100 μm in x,y and z dimensions) • Suitable for clinical translation	• Exposure to ionizing radiation which can cause DNA damage • Not suitable for soft tissues
Radionuclide imaging	• Only low doses of labels need to be employed due to the high sensitivity of the probes • Good tissue penetration of the probe • Suitable for clinical translation • Fair sensitivity (10^–8^ to 10^–9^μm/L)	• Exposure to ionizing radiation which can cause DNA damage • Half-life of the probe must be considered

*3D* three-dimensional, *DHM* digital holographic microscopy, *NIR* near infrared, *nsOCT*, *OCPM*, *OCT* optical coherence tomography, *OCM* optical coherence microscope, *ODT*, *PAI* photoacoustic imaging, *PALM* photo-activated localization microscopy, *QPM* quantitative phase microscopy, *SESF* spectral encoding of the spatial frequency, *srSESF*, *STED* stimulated emission depletion, *STORM* stochastic optical reconstruction microscopy, *nsOCT* nano-sensitive optical coherence tomography, *OCPM* optical coherence phase microscopy, *ODT* optical doppler tomography, *srSESF* super-resolution spectral encoding of spatial frequency

Future directions should focus on multimodality imaging approaches that can combine the strength of each modality for a comprehensive detection and minimise potential drawbacks of using the imaging technique alone. Developing biodegradable contrast agents and multimodal contrast agents is another future development direction. The cytotoxicity and potential toxicity can be effectively reduced using degradable contrast agents by facilitating the clearance of the contrast materials [109]. Future directions of microscopic-related technologies will more than likely be in parallel with the development of advanced label-free imaging techniques and those which employ non-toxic cellular contrasting agents. Future development of imaging modalities for stem cell study should be focused on specific needs for different applications, but all applications would benefit from increased resolution, sensitivity, and reduced toxicity.

## Conclusions

The vast array of technologies discussed above that are available to clinical and scientific researchers in the field of regenerative medicine allow multiple different elucidating conclusions to be drawn from imaging or analysing the tissue under study. The development of multimodal techniques which have the capacity to employ more sensitive, accurate, and less toxic labels to image deeper into the innate tissue in vivo will in time greatly further discoveries in this field. In relation to stem cell tracking for regenerative medicine, the availability of imaging systems (combination of hardware and cell-labelling strategy) will determine the cell-labelling strategy, with each approach having advantages and disadvantages. In general, the ideal system should have high spatial (ability to resolve single cells) and temporal resolution, contrast, sensitivity (detect small numbers of cells), be relatively easy of use, and be inexpensive. No imaging strategy will tick all the boxes; however, the current trend towards multimodal imaging can exploit one system’s advantages while negating the disadvantages of another.

## Abbreviations

3D: Three-dimensional
Au: gold
CLARITY: clear lipid-exchanged acrylamide-hybridized rigid imaging/immunostaining/in situ-hybridization-compatible tissue hydrogel
CT: computed tomography
DHM: digital holographic microscopy
DS: dielectric spectroscopy
GFP: green fluorescent protein
HD-OCT: high-definition optical coherence tomography
hIPSC: human induced pluripotent stem cell
hMSC: human mesenchymal stem cell
hPSC: human pluripotent stem cell
iRFP: near-infrared fluorescent protein
IRM: interference reflection microscopy
MR: magnetic resonance
MSC: mesenchymal stem cell
NIR: near infrared
NIS: sodium iodide symporter
NP: nanoparticle
NR: nanorod
OI-RD: oblique-incidence reflectivity difference
OCM: optical coherence microscope
OCT: optical coherence tomography
PAI: photoacoustic imaging
PALM: photo-activated localization microscopy
PET: positron emission tomography
PT-OCT: photothermal optical coherence tomography
QPM: quantitative phase microscopy
SESF: spectral encoding of the spatial frequency
SiGNR: silica-coated gold nanorod
SR-OCT: spectral radar optical coherence tomography
SDOCT: spectral domain optical coherence tomography
SPECT: single-photon emission computed tomography
SSEA1: stage-specific embryonic antigen 1
STED: stimulated emission depletion
STORM: stochastic optical reconstruction microscopy
SWNT: single-walled carbon nanotube
US: ultrasound
